# Updates on Caries Risk Assessment—A Literature Review

**DOI:** 10.3390/dj12100312

**Published:** 2024-09-29

**Authors:** Toby Cheuk-Hang Ng, Bella Weijia Luo, Walter Yu-Hang Lam, Aylin Baysan, Chun-Hung Chu, Ollie Yiru Yu

**Affiliations:** 1 Faculty of Dentistry, The University of Hong Kong, Hong Kong SAR, China; tobyng@connect.hku.hk (T.C.-H.N.); bellaluo@hku.hk (B.W.L.); retlaw@hku.hk (W.Y.-H.L.); chchu@hku.hk (C.-H.C.); 2 School of Medicine and Dentistry, Queen Mary University of London, London EC1M 6BQ, UK; a.baysan@qmul.ac.uk

**Keywords:** dental caries, caries risk assessment, cariology, preventive dentistry, oral health

## Abstract

This narrative review aims to provide an update on caries risk assessment (CRA) and the available CRA tools. CRA can be used to monitor the status of oral care, as well as for documentation and research purposes. Caries risk is determined by the interplay of risk and protective factors. Common risk factors include suboptimal oral hygiene practices, previous caries experience, low plaque acidity, frequent snacking, cariogenic diet, enamel defects, reduced salivary flow, polypharmacy, and radiotherapy experience. Caries risk can be reduced through some preventive measures, such as the use of fluoride, gum chewing, dental sealants. The CRA process can be categorized as single or multifactor tools. Single-factor CRA tools include diet analysis questionnaires, cariogenic bacteria testing and saliva testing kits, whereas multifactor CRA tools encompass Caries Management by Risk Assessment (CAMBRA), Cariogram, and PreViser. Some CRA tools may quantify risk and protective factors to compute caries risk. Additionally, they can generate visual and educational presentations that foster success in oral care. Clinicians consider CRA beneficial for developing personalized care plans. However, the literature fails to reflect this and reveals limited studies supporting its use as evidence-based practice for caries management.

## 1. Introduction

Dental caries is prevalent worldwide, affecting individuals of all age groups. The contemporary philosophy of dental caries management embraces the principle of minimal intervention dentistry (MID). MID places emphasis on keeping the teeth healthy throughout one’s life personalized prevention and management plans, and advocates for noninvasive and/or minimally invasive treatment [1]. Caries risk assessment (CRA) is one of the five elemental strategies in MID that should be employed throughout a person’s life [2].

Incorporating CRA into a personalized management plan has been recognized as highly beneficial [2]. Caries risk is never static but changes throughout one’s life. An accurate evaluation of caries risk allows for the customization of the personalized management plan for dental caries and an effective control of dental caries, particularly for patients with high caries risk [3]. Therefore, CRA is an essential element of dental treatment planning and provides valuable guidance for clinical practice.

Identifying the risk and protective factors is the first step for the CRA. An individual’s caries risk is affected by multiple factors including caries risk and protective factors presented either locally or systemically. Caries risk factors are conditions that enhance the possibility of developing dental caries, while caries protective factors are conditions that reduce the possibility of caries development. Numerous studies have been conducted to confirm the factors that will affect the development of dental caries [4,5,6].

It is a complicated process to determine one’s caries risk. Multiple-factor CRA tools have been, therefore, developed [7,8,9]. These tools include protective factors for evaluation and are often available as software to compute caries risk. The software has the merits of providing a comprehensive analysis and personalized CRA. Additionally, clinicians can use software of CRA tools for visual presentation and educational tools to promote the oral care of their patients.

Although CRA tools are taught in dental schools and used by clinicians, the adoption of CRA in daily clinical practice is limited [10,11]. Lack of evidence has been observed in the implementation of CRA tools as part of patient care. This review aims to provide an update on CRA and the available tools and to explore the evidence to support the CRA tools’ use for caries management.

## 2. Literature Search

### 2.1. Search Strategy

A systematic search was conducted in three databases (PubMed, Web of Science, and Scopus databases) to identify all relevant studies published before 30 December 2023, in the English language. The search strategies were (caries risk assessment) AND ((validation) OR (sensitivity) OR (specificity)).

### 2.2. Study Selection

The title and abstract of the identified publications were independently screened by two reviewers. Full texts of all potentially relevant publications were obtained and independently reviewed after the screening.

The inclusion criteria of this review were clinical studies (randomized clinical trials, controlled clinical trials, case–control study, cross-sectional study, and cohort study) that displayed the concept of caries risk or caries risk assessment tool. The goal was to identify studies that reported the sensitivity and specificity of the multiple caries risk assessment tools. The exclusion criteria were the studies that were not written in English or were irrelevant to dental caries or caries risk assessment tool.

The process was independently conducted by two reviewers. If no consensus was reached, a third reviewer was consulted for the final decision.

### 2.3. Results

**Figure 1:**
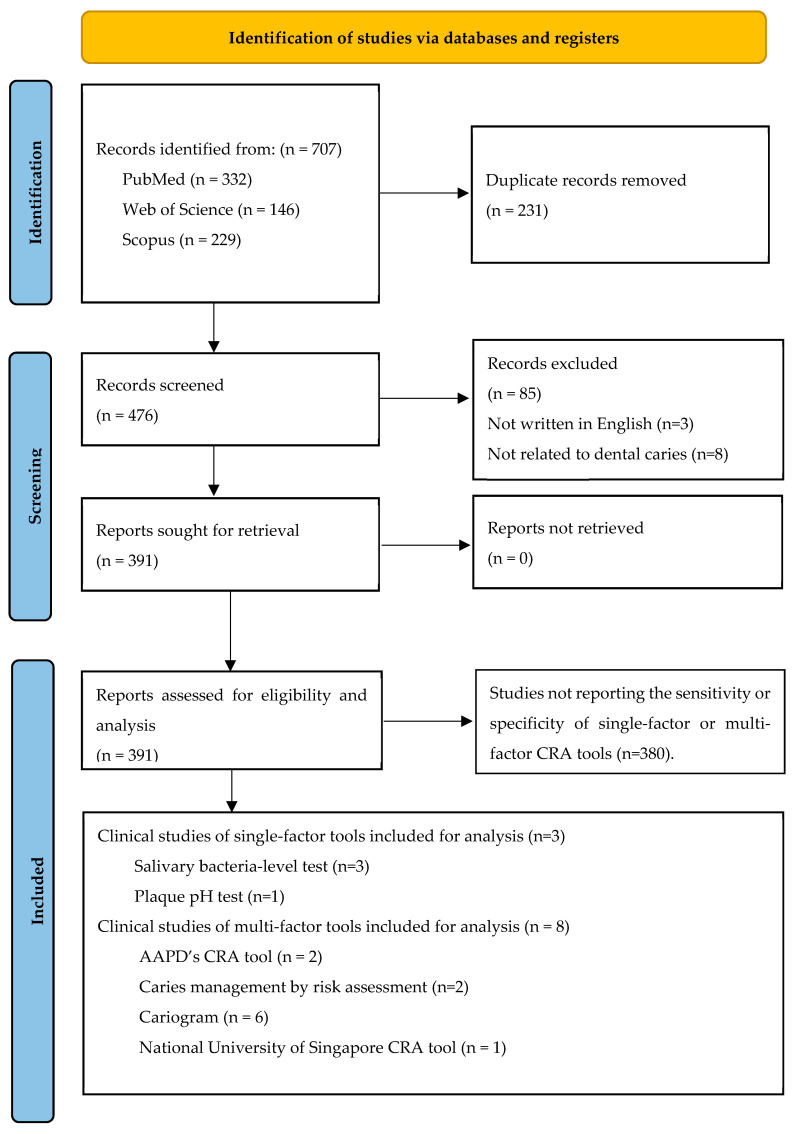
Flow diagram of the literature search.

A total of 707 records written in English were screened. Study screening and data extraction were independently performed by two reviewers (T.N. and O.Y.). The study selection process is presented in Figure 1. Among these studies, 231 duplicates were removed. After full-text review, 3 cross-sectional studies [12,13,14] about single factor-tools and 8 prospective cohort studies [15,16,17,18,19,20,21,22] about multifactor tools were eligible to be included in this review. Among the studies about single-factor tools, three of them were about the salivary bacteria-level test and one of them was about the salivary pH test. One of the studies investigated both types of tests. For the studies of multiple-factor tool, two studies were about CRA tools of American Academic of Pediatric Dentistry (AAPD), six studies were about caries management by risk assessment, two studies were about Cariogram, and one study was about National University of Singapore CRA tool. One of the studies investigated four caries risk assessment tools. The process is shown in Figure 1.

## 3. Factors Affecting Patients’ Caries Risk Levels

Caries risk and protective factors affect caries risk level of the patients, which indicates the likelihood of a patient developing dental caries in future. The following are risk and protective factors that have been reported in the literature.

### 3.1. Risk Factors

#### 3.1.1. Oral Environmental Risk Factors

##### Past Caries Experience

Past caries experience is an important risk factor to be considered [23]. A systematic review [24] concluded that baseline caries prevalence was the most accurate factor to predict future caries incidence in all age groups based on a multivariate model. Other studies supported the evidence that the past caries experience was associated with the development of dental caries in future [25,26], and even related to the degree of untreated caries [27].

##### Salivary *Streptococcus mutans* (*S. mutans*) Level

The untreated caries is positively related to the salivary *S. mutans* [27]. When compared to children without *S. mutans*, children with higher levels of salivary *S. mutans* were more likely to experience caries increments [28,29].

##### Plaque pH

The evidence supported that lower plaque pH indicated a high caries risk [30]. Studies [30,31] supported that participants with more decayed tooth surfaces were found to have lower plaque pH.

##### Malocclusion

The evidence about the impact of malocclusion on the caries risk was inconclusive among studies. One systematic review [32] supported the association between malocclusion and the risk in increasing dental caries. Contrast to this, another systematic review [33] found that the evidence was absent regarding the association between malocclusion and oral health.

##### Enamel Defects

Developmental defects of enamel contribute to a high caries risk [34]. Two systematic reviews [35,36] supported that enamel defects were the risk factor associated with early childhood caries (ECC), due to its low quality in tissue.

#### 3.1.2. Personal Behavior Factors

##### Diet

Evidence consistently supported the association between diet and caries. Diets, such as sugar intake and snacking habits, were found to be an important risk factor in caries development [37]. Individuals who had the habits of high sugar-sweetened beverage consumption [38,39], frequent intake of snacking [40,41], and consuming free sugar [42,43] were more likely to increase caries risk.

##### Maternal Conditions

The oral status, social status, and behavior of a mother could be considered to predict the caries risk of their child. Children whose mothers had low socioeconomic status, poor oral health status [44], or had high salivary *S. mutans* levels [45] or smoking habits [41,46] had a higher risk of developing dental caries.

#### 3.1.3. Systemic Factors

##### Radiotherapy

Head and neck radiotherapy targeting salivary glands and hard tissues potentially increases the patients’ caries risk. Radiation can directly affect the tooth structure. The micromorphology, mechanical properties, and chemical composition of dental hard tissue could be altered [47,48]. Radiation therapy may damage the normal function of salivary glands, resulting in reduced salivary flow (hyposalivation), which is a common adverse effect of the head and neck and leads to a higher caries risk [49,50].

##### Diabetes Mellitus

Diabetes mellitus increases the patients’ caries risk. The buffering capacity of saliva was found to be low in children with Type 1 diabetes [51]. Patients with Type 1 diabetes [52] and Type 2 diabetes [53] were found to be related to a high caries risk.

##### Sjögren’s Syndrome

Evidence supported that patients with Sjögren’s syndrome had a high caries risk. Most patients had hyposalivation due to Sjögren’s syndrome [54]. The reason is not yet determined.

##### Obesity

No definite conclusion on the correlation between obesity and caries risk has been reached. Some systematic reviews [55,56] and studies [57,58] supported that participants with obesity had a higher risk of developing dental caries, while other studies [59,60,61] failed to support obesity as a risk factor of developing dental caries.

##### Stress

The evidence of the association between emotional distress and dental caries was limited. It was previously reported that patients with emotional distress were more likely to have dental caries [62], which may be explained due to suboptimal plaque control [62].

### 3.2. Protective Factors

#### 3.2.1. Oral Environmental Protective Factors

##### Saliva

Saliva with high pH, high flow rate, and buffering capacity can protect an individual from developing dental caries in the way of neutralizing the lactic acid produced by cariogenic bacteria [63]. Furthermore, antibacterial compounds such as lysozyme and lactoferrin can be found in saliva [64], which can also shield a tooth from caries.

##### Fluoride

The use of fluoride mouth rinse can largely reduce the caries increment [65]. Toothbrushing with fluoridated toothpaste reduced the caries increment when compared to nonfluoridated toothpaste [66]. Moreover, topical fluoride application has also been proven to reduce the incidence of dental caries in several studies [67,68].

##### Chewing Gum

Chewing sugar-free gum is a protective factor in caries development [69], which could reduce the load of *S. mutans* [70,71], especially with xylitol-containing gums [72].

##### Probiotics

Probiotics can reduce the caries risk of a patient. It was reported that probiotic supplements can reduce the caries experience of the participants [73]. Probiotics were also shown to reduce the salivary level of *S. mutans* and prevent caries in preschool children [74]. This can be explained by the effect of inhibiting gene expression of *S. mutans* and biofilm formation [75].

##### Dental Sealant

Dental sealant can effectively reduce the risk of dental caries [76]. A systematic review concluded that the caries risk of occlusally sealed teeth can be reduced by up to 85% within 84 months [77].

#### 3.2.2. Systemic Protective Factors

##### Systemic Fluoride

Fluoridated water and fluoride supplements are two methods of systemic fluoride intake. They are shown to significantly reduce the incidence of dental caries [78,79]. Fluoride supplements can be considered for children with high caries risk, but not as the first option [80]. However, one should be careful when using fluoride supplements, as excessive fluoride intake can cause dental fluorosis.

## 4. Caries Risk Assessment (CRA) Tools

CRA tools are used to identify the caries risk of a particular patient. Based on the number of factors assessed, they can be classified as single-factor or multiple-factor CRA tools.

### 4.1. Single-Factor CRA Tools

A single-factor CRA tool measures only one parameter to determine the caries risk of the patient. It is simple to conduct. However, when compared to multiple-factor CRA tools, it is less comprehensive, and no management recommendations would be given along with these tools. The sensitivity and specificity of salivary bacteria-level test and plaque pH test are listed below in Table 1.

#### 4.1.1. Salivary Bacteria-Level Test

Testing the level of cariogenic bacteria in saliva is a common approach in assessing the caries risk of in the patient. It is easy to use and to communicate with patients [81]. The Saliva-Check mutans kit (GC, Tokyo, Japan) can identify patients with high *S. mutans* counts, and the sensitivity and specificity reported in one study [12] were 88% and 90%, respectively, and in another study were 88% and 75%, respectively [14]. However, another low specificity, 25%, was reported in another clinical study [13]. The Caries Risk Test (Ivoclar Vivadent, Schaan, Liechtenstein) determining the bacterial counts with a culture medium, and Cariscreen Caries Susceptibility Test (Oral BioTech, New York, NY, USA) using a meter to detect bacterial loads yielded sensitivity and specificity exceeding 90% [14].

#### 4.1.2. Salivary Property Test

Salivary pH, salivary flow rate, and salivary capacity can be measured with corresponding test kits to assess the patient’s caries risk. However, the use of saliva in assessing the caries risk of a patient is contradictory. Some studies reported that higher pH was associated with low caries risk [82,83]. However, there was conflicting evidence as these tests are unreliable in determining caries risk [84] or weakly linked to recent dental caries experience [85]. Further assessment is required to reach a conclusion.

#### 4.1.3. Salivary Immunoglobulin Level

The use of immunoglobulin levels as an indicator of caries risk is controversial. One systematic review [86] supported the evidence of a positive relationship between salivary immunoglobulin level and caries-active individuals. On the contrary, another systematic review [87] reported no conclusions on the relationships between salivary immunoglobulin and dental caries.

#### 4.1.4. Plaque pH Test

A pH test kit, Plaque-check pH kit (GC, Tokyo, Japan), was used and reported with 72% sensitivity and 55% specificity, respectively [12]. The comparison between Cariview (AIOBIO, Seoul, Republic of Korea) and Dentocult SM(^®^) test (Orion Diagnostica, Espoo, Finland) showed a stronger correlation between plaque pH and caries risk [30].

### 4.2. Multiple-Factor CRA Tools

Multiple-factor CRA tools require medical and dental history provided by the patients and the clinical information obtained from clinical examination. These tools are more complex and comprehensive than the single-factor risk assessment tools. In terms of the mode of data processing, they can be divided broadly into form- and algorithm-based tools. The common multiple-factor CRA tools are introduced in this section and their accuracy based on the previous report is summarized in Table 2.

#### 4.2.1. Form-Based CRA Tools

Form-based CRA tools list the caries risk factors or predictors and use them to assess caries risk. Instructions on how to use the form are normally provided along with the forms. Most are readily accessible and are free of charge.

##### American Academy of Pediatric Dentistry’s Caries Risk Assessment Tool (AAPD-CAT)

The AAPD provides separate form-based CRA tools for children and adolescents [7]. One is for those aged 0–5 years old and another is for patients aged above 6. The forms are available on the website of the AAPD with the latest update in 2022. Social, behavioral and medical risk factors (hypo-salivary medication and frequent snacking), clinical factors (visible plaque), protective factors (topical fluoride usage), and disease indicators (white spot lesions) are taken into account for CRA.

##### American Dental Association’s Caries Risk Assessment Tool (ADA-CAT)

The ADA provides separate form-based CRA tools for children and adolescents. One is for those aged 0–6 years old and another is for patients aged over 6. The forms are available on the website of the ADA. The latest update was in 2011. Conditions (fluoride exposure and sugary food uptake), general health conditions (special health care needs), and clinical conditions (visible plaque) are taken into consideration.

##### Caries Management by Risk Assessment (CAMBRA)

One study [88] explored refining ADA-CAT to help the healthcare provider determine the caries risk of individuals, and they updated the form in 2021. Health and lifestyle factors are compared against protective factors to determine the caries risk. CAMBRA can be used free of charge. However, permission must be obtained before any commercial use.

##### International Caries Classification and Management System (ICCMS™) and CariesCare International (CCI) System

The ICCMS™ enables dentists to plan, manage, and review caries in clinical practice according to the caries risk and caries status [8]. A 4D protocol was proposed according to the plan, including “Determining the patient’s caries risk”, “Detecting and assessing caries lesions”, “Deciding the treatment plan”, and “Do the intervention”. CRA was involved in the first two steps of the 4D protocol. Free ICCMS™ guide can be found on the ICCMS™ website. An upgraded system CariesCare International (CCI) system was derived from ICCMS™ in 2019 [89].

#### 4.2.2. Algorithm-Based CRA Tools

Algorithm-based CRA tools are used to assess caries risk through a programme by inputting the data retrieved from clinical examinations and history. Cariogram, MysmileBuddy, Risk Assessment Tool (PreViser), and the National University of Singapore Caries Risk Assessment Tool (NUS-CRAT) are examples of algorithm-based CRA tools. Patient–dentist communication may be enhanced because of the graphic presentation of the caries risk using these tools. It should be noted that most algorithm-based CRA tools require an extra subscription fee for the purchase of software or registration.

##### Cariogram

Cariogram is an algorithm-based software predicting the caries risk of a patient with a graphical presentation [90]. It can generate the caries risk of patients according to the information on different factors such as caries experience, diet frequency, and salivary buffering capacity. No treatment suggestions will be given in this software after having the results of caries risk assessment. Cariogram can be downloaded from the website of the Malmö University with a manual and can be used for free in educational or noncommercial activities.

##### MySmileBuddy (MSB)

MSB was developed to be an interactive CRA tool for children and their parents for educational purposes [91]. CRA can be performed by the patients themselves by inserting data without clinical findings. Diet scores and comprehensive risk scores can be calculated. Concerning the level of caries risk, MSB provides suggestions for intervention to reduce the caries risk.

##### PreViser Risk Assessment Tool (PreViser)

PreViser (Denplan, London, United Kingdom) is designed for members of Denplan, which is a company specializing in providing dental payment plans and serving UK consumers. Generally, positive comments were collected from dentists included in the pilot studies [92,93].

##### National University of Singapore Caries Risk Assessment Tool (NUS-CRAT)

NUS-CRAT was created by the National University of Singapore. This tool is not available online. Patients undergoing this tool would be categorized into five caries risk groups [94].

#### 4.2.3. Accuracy of Multiple-Factor CRA Tools

The accuracy of form-based and algorithm-based CRA tools that were investigated is presented in Table 2.

For form-based CRA tools, AAPD-CAT for patients under 6 years old was reported as a high-sensitivity and low-specificity tool to access caries risk in previous studies, with a sensitivity of 100% and a specificity of 5% in one study [15], and a sensitivity of over 90% and a specificity of below 10% in another study [16]. The accuracy of AAPD-CAT in patients aged 6 or above is yet to be determined.

The accuracy of CAMBRA for children under 6 or below was inconsistent in studies. One study reported a sensitivity of 84% and a specificity of 63% [15], while another study reported a sensitivity of 48% and a specificity of 80% [17]. For participants aged above 6 years old, CAMBRA showed the ability to predict caries risk, which supported its use in patient management [95]. CAMBRA, with its ability to visualize the caries risk, was also used to prevent dental caries by improving participants’ oral health behaviors [96]; however, there is lack of robust evidence due to the design and possible bias of the included studies [97].

The accuracy of the ICCMS™ system has not been investigated yet. Compared with CAMBRA regarding the effectiveness of caries prevention, the ICCMS™ system showed no difference in the ability to prevent dental caries [98].

For algorithm-based CRA tools, the accuracy of Cariogram was inconsistent in studies with no sufficient evidence concluded by one systemic review [99]. A one-year study [15] reported a sensitivity of 65% and a specificity of 79%. Another study [18] reported a sensitivity of over 90% and a specificity of below 30% in the high caries risk group, 61% and 71% in the moderate caries risk group, and 90% and 34% in the low caries risk group. On the contrary, one study [19] reported a sensitivity of 12% and a specificity of 100% in the low caries risk group, while 99% and 24% in the high caries risk group.

The accuracy of NUS-CRAT was reported by one study [15] with a sensitivity of 78% and a specificity of 85%.

No study investigating the accuracy of MSB and PreViser tool were available.

## 5. Discussion

This review provided an update on CRA and the CRA tools and explored the evidence to support CRA tools’ use for caries management.

Regarding the comparison of single-factor and multiple-factor CRA tools, both of them play essential roles in clinical practice with advantages and disadvantages. Single-factor tools offer simplicity, quick assessment, and cost-effectiveness; however, limited scope and potential for false conclusions may restrict the usefulness in some cases, where recommendations are needed. Multiple-factor tools provide a comprehensive assessment and personalized approach, which can lead to more effective treatment plans and improved patient education. However, their complexity, time-consuming nature, and cost may pose challenges for some dental professionals. The choice of single-factor and multiple-factor CRA tools mostly depends on the needs of clinical practice.

In the context of comparing various multifactorial CRA tools, the accuracy of form-based and algorithm-based CRA tools has been investigated to predict caries risk. While there is limited evidence available to draw a definitive conclusion, some studies have provided insights into their comparative performance. For instance, a two-year clinical study [100] found that the Cariogram demonstrated higher validity than the AAPD-CAT. Conversely, another three-year study [18] revealed that the Cariogram did not outperform form-based CRA, which relies on past caries experience.

The potential reasons for variability in tool accuracy were complicated. An accurate evaluation of an individual’s caries risk enables dental professionals to customize personalized management plans and effectively control dental caries. The caries development is dynamic and changes throughout a person’s life due to various factors, such as oral environmental condition, personal behavior factors, and systemic factors. It is difficult to predict a long-term outcome based on those short-term assessment results.

To improve the accuracy of the tools, it is very important to train the dental professionals better before utilizing the tools. As a result, the inconsistent findings emphasize the need for additional research to delve deeper into the comparative accuracy of form-based and algorithm-based CRA tools.

To gain a clearer understanding of the effectiveness of the CRA tools and provide more concrete guidance for dental professionals, further studies exploring this topic are warranted. These studies should ideally account for the varying expertise and training of the dental professionals, as well as the diverse patient populations they serve, to ensure a comprehensive evaluation of these assessment tools.

In the realm of m-health, the utilization of teledentistry has proven to be advantageous for dental care, especially in the era of COVID-19 [101,102]. Algorithm-based CRA tools serve as an essential aid for dentists in documenting and monitoring patient conditions, thus playing a pivotal role in the advancement of teledentistry. These tools, powered by data-driven algorithms, facilitate the evaluation of a patient’s risk of developing dental caries. This enables the provision of personalized preventive care and paves the way for early interventions. Dentists can leverage these tools to prioritize patients based on their caries risk, allowing for an efficient allocation of resources. This strategy focuses attention on patients requiring more intensive care and monitoring, optimizing the use of limited resources, especially in underserved areas. Additionally, these algorithm-based CRA tools can be integrated with other teledentistry technologies like digital imaging and electronic health records. This integration facilitates the remote monitoring of a patient’s oral health, empowering dental professionals to track alterations in a patient’s caries risk over time and adjust treatment plans accordingly.

This review’s strength is rooted in its all-encompassing and meticulous synthesis of the topic in focus. By thoroughly exploring the diverse aspects and viewpoints associated with CRA and CRA tools, the review equips researchers with a wide-ranging understanding of the field. This encompasses both the fundamental principles and the latest developments, ensuring a well-rounded and current knowledge foundation on the subject matter.

The limitations of this review are primarily twofold. Firstly, this review may be subject to publication bias. This implies that studies with positive results are more likely to be published and included in this review, potentially skewing the overall interpretation and conclusions. Secondly, this review only includes publications that are written in English. This language restriction may exclude relevant studies published in other languages, potentially leading to an incomplete overview of the global research on the topic. These limitations could affect the comprehensiveness and representativeness of the review’s findings.

Caries risk refers to a patient’s probability of developing dental caries in the future, which influences the management plan tailored for the patient [95]. Assessing a patient’s caries risk involves considering their dental and medical history, as well as clinical findings. The interplay between risk factors and protective factors determines caries risk. Risk factors, such as past caries experience, low plaque pH, improper diet, enamel defects, diabetes, Sjögren’s disease, and radiotherapy experience, have been found to be associated with an increased caries risk. On the other hand, protective factors like high salivary buffering capacity, fluoride application, gum chewing, probiotics, and dental sealant application may decrease a patient’s caries risk level.

Various CRA tools are available to evaluate a patient’s caries risk based on single or multiple relevant factors. However, there is currently insufficient evidence to establish the superiority of any particular tool. This highlights the need for further research on the accuracy of existing CRA tools and the development of innovative tools to assess caries risk more effectively.

## 6. Conclusions

Caries risk assessment is beneficial for developing patient’s dental care plans. However, the evidence is limited for supporting its use as evidence-based practice for caries management. More well-designed studies are needed to assess the sensitivity and specificity across different CRA tools. Professional training should be provided to dental health care providers before utilizing CRA tools for clinical practice.

## Figures and Tables

**Table 1:** The sensitivity and specificity of single-factor caries risk assessment tools.

Author (Year)	Brand	Age (Year)	Sample Size	Sensitivity (%)	Specificity (%)
**Salivary bacteria-level test**					
Strickland et al. (2017) * [12]	Saliva-Check Mutans (GC, Tokyo, Japan)	Adult: 25–35 Children: 3–10	Adult: 20 Children: 25	88	90
Voelker et al. (2018) [13]	Saliva-Check Mutans (GC, Tokyo, Japan)	NA	53	88	25
Babu et al. (2019) [14]	Saliva-Check Mutans (GC, Tokyo, Japan)	9–14	25	88	75
	Caries Risk Test (Ivoclar Vivadent, Schaan, Liechtenstein)			93	92
	Cariscreen Caries Susceptibility Test (Oral BioTech, New York, United States)			92	92
**Plaque pH test**					
Strickland et al. (2017) * [12]	Plaque-check pH kit (GC, Tokyo, Japan)	Adult: 25–35 Children: 3–10	Adult: 20 Children: 25	72	55

* The same publication.

**Table 2:** The sensitivity and specificity of multiple-factor caries risk assessment tools.

Author (Year)	Follow-Up Duration (Year)	Age at Baseline (Year)	Final Sample Size	Sensitivity (%)	Specificity (%)
**American Academy of Pediatric Dentistry’s Caries Risk Assessment Tool**					
Gao et al. (2013) * [15]	1	3	485	100	3.6
Agouropoulos et al. (2019) [16]	2	2–5	146	94	7
**Caries Management by Risk Assessment Tool**					
Gao et al. (2013) * [15]	1	3	485	84	63
Sudhir et al. (2016) [17]	2	12–13	72	48	80
**Cariogram**					
Gao et al. (2013) * [15]	1	3	485	65	79
Peterson (2015) [18]	3	19	982	Low risk: 89 Moderate risk: 61 High risk: 26 Very high risk: 12	Low risk: 34 Moderate: 71 High risk: 91 Very high risk: 95
Dou (2018) [19]	2	18–29	192	Very low risk: 12 Low risk: 26 Moderate risk: 56 High risk: 84 Very high risk: 99	Very-low-risk: 100 Low risk: 91 Moderate risk: 80 High risk: 56 Very high risk: 24
Kim et al. (2018) [20]	NA	10–18	171	61	85
Birpou et al. (2019) [21]	2	2–5	154	68	59
Dolic et al. (2020) [22]	4	0	80	Very low to moderate risk: 61 Moderate to very high risk: 82	Very low to moderate risk: 91 Moderate to very high risk: 65
	4	20–42	80	Very low to moderate risk: 54 Moderate to very high risk: 81	Very low to moderate risk: 69 Moderate to very high risk: 58
**National University of Singapore Caries Risk Assessment Tool**					
Gao et al. (2013) * [15]	1	3	485	78	85

* The same publication.

## Data Availability

No new data were created or analyzed in this study. Data sharing is not applicable to this article.
